# Roles of reactive oxygen species, storage compounds, and endogenous hormones in regulating somatic embryogenesis in blue spruce (*Picea pungens* Engelm.)

**DOI:** 10.3389/fpls.2025.1646777

**Published:** 2026-01-12

**Authors:** Shigang Chen, Fang Gao, Xi Cao, Huanyu Dong, Yuchen Zhan, Li-Hua Zhu, Jing Tao

**Affiliations:** 1 Institute of Forest Research, Jilin Academy of Forestry Sciences, Changchun, China; 2 Department of Plant Breeding, Swedish University of Agricultural Sciences, Lomma, Sweden

**Keywords:** blue spruce, endogenous hormone, *Picea pungens* Engelm., plant growth regulator, ROS, somatic embryogenesis

## Abstract

Four freshly established cell lines of blue spruce (*Picea pungens* Engelm.), with different capacities for somatic embryogenesis (SE), were studied to assess their SE formation and maturation, aiming at identifying key factors responsible for the variation in SE potential. We examined dynamic cytological and physiological changes, including morphology and structure, storage substances, antioxidant enzyme activities and endogenous hormone levels. The results showed that cell lines 202, 201, 211 with SE capacity exhibited active metabolic processes and significant increases in superoxide dismutase (SOD) and peroxidase (POD) activities, total protein (TP) and malondialdehyde (MDA) contents across developmental stages. In contrast, the cell line lacking SE capacity showed low or unchanged enzyme activities. These findings suggest that SOD, POD, MDA and TP can serve as physiological markers for identifying SE capacity. During SE development and maturation, endogenous hormone levels (GA, IAA, ABA) remained unchanged in the non-embryogenic cell line. However, in highly embryogenic line, hormone levels generally increased and then decreased, while lines with intermediate and weak SE showed a steady increase. Our findings provide critical insights into optimizing culture media formulations to enhance blue spruce SE maturation, thereby improving efficiencies of elite genotype selection and large-scale clonal propagation. Such advancements are essential for meeting the growing demand for high-quality planting materials in landscape applications and forestry production. Nevertheless, the underlying molecular mechanisms by which reactive oxygen species (ROS) influence SE maturation capacity, as well as the synergistic regulatory interactions among endogenous hormones during different embryo developmental stages, remain insufficiently understood and warrant further investigation. Furthermore, the technological framework and regulatory patterns identified in this study are not limited to blue spruce; the physiological markers and developmental cues elucidated herein may serve as valuable references for SE research in coniferous species. To validate the practical applicability of this approach, comprehensive field trials are required to evaluate the growth performance, adaptability, and stability of SE-derived plants under natural environmental conditions.

## 1 Introduction

The needles of blue spruce (*Picea pungens* Engelm.) show silver-blue color throughout the year, which is of high ornamental value and is a rare colorful foliage species in gardens that has been widely planted as an ornamental tree in North America and European countries (Tao et al., 2021; Fechner, 1984). Blue spruce, introduced into northeastern China since 2000, has been widely used as an important ornamental plant, and seed gardens have been established (Tao et al., 2021). Currently, blue spruce is commonly propagated by seeds in China, but the number of seedlings produced is limited due to the high prices of seeds and seedlings (Sun and Jia, 2010). Moreover, seed propagation results in substantial genetic and phenotypic variation in important traits such as needle color (Tao et al., 2021), morphology (Rodríguez-Calcerrada et al., 2022), and structure and growth rate (Li et al., 2023). This leads to ununiform trees in commercial production, which is undesirable (Tao et al., 2021; Liu et al., 2023). On the other hand, the high genetic diversity generated by seed propagation may facilitate adaptation to biotic and abiotic stresses, thereby contributing long-term ecosystem resilience and sustainability. In contrast, somatic embryogenesis (SE) produces genetically identical individuals, enabling clonal multiplication of selected superior genotypes while preserving the key traits and providing efficient, uniform large-scale propagation (Savane et al., 2023). This makes SE particularly suitable for commercial production in controlled environments, such as nurseries and urban areas, whereas seed-based propagation can be prioritized in wild and large-scale afforestation to maintain genetic diversity. On the other hand, plantations established from a limited number of SE-derived genotypes may exhibit reduced genetic diversity and can therefore be more vulnerable at the population level to emerging pathogens and environmental extremes (Savane et al., 2023).

In contrast, somatic embryogenesis offers possibilities to use the same elite seed to produce a large number of embryonic asexual lines (Guo et al., 2020), producing a large number of genotypically identical plants in a short period of time (Chen et al., 2021; Zhang et al., 2022; Li et al., 2023), which ensures the consistency of plant genetic traits and is of great research value for the expansion of blue spruce. Compared to somatic embryo propagation, seed propagation has lower initial costs but results in greater genetic variations among offspring, leading to increased screening and management costs in later stages. In contrast, somatic embryo propagation preserves desirable traits, offers high propagation efficiency, and enables the rapid, large-scale production of uniform asexual lines. Although it involves significant upfront investment due to the need for specialized equipment, costs decrease with scale, and its feasibility improves as demand grows. The need for uniform, high-quality seedlings for afforestation and ecological restoration drives strong demand for superior planting materials in the forestry sector.

Markers of plant embryogenesis are an important aspect of modern plant breeding (Jiménez and Bangerth, 2001), and early identification of somatic embryogenic competence is important to ensure subsequent steps in the somatic embryogenesis (SE) process. During the proliferation stage of embryonic callus (EC), the efficiency of SE can be significantly improved by eliminating non-embryonic callus (NEC) without SE capacity and accurately screening cell lines with excellent SE capacity. The macroscopic morphology of the callus tissues could not be used as a criterion to accurately judge NEC from EC. Currently many studies have been conducted mainly on the cytological differences between EC and NEC, as well as on the physiological and biochemical changes during somatic embryo (SE) culture (Klimaszewska et al., 2000; Zhang et al., 2021; Heringer et al., 2015; Li et al., 2015).

However, studies on physiological mechanisms underlying differences in SE maturation capacity in coniferous trees have been much less reported (Businge et al., 2012). SE formation is driven by spatiotemporally regulated expression of SE-related genes, leading to quantitative and qualitative proteomic changes that are closely linked to cellular function, tissue morphology, and the plant’s physiological and biochemical status. The organism provides the necessary energy for protein synthesis by consuming saccharides, and the transformation and accumulation between starch (ST), soluble sugar (SS), and soluble total proteins (TP) play an important role in somatic embryogenesis (Neves et al. (2003) and Mahmud et al. (2015) analyzed the metabolomic differences between EC and NEC in sugarcane, including fat, soluble proteins, amino acids, carbohydrates, organic acids, phenolics and polyamines, and the results showed the embryogenic potential of callus was determined by a variety of metabolites. During plant growth and development, ST and SS act as energy storage substances to provide energy for plant metabolism (Peng et al., 2020). SE is dependent on carbohydrate metabolism, which can provide energy and carbon sources for biosynthesis of regulatory proteins during SE (Noah et al., 2012). Therefore, recognizing the morphological and physiological differences between EC and NEC is essential for improving the SE technology system (Peng et al., 2020).

Reactive oxygen species (ROS) are oxygen-containing metabolites and derivatives produced during the metabolic processes of living organisms (Sofo et al., 2010), including superoxide anion radicals (·O2-), hydrogen peroxide (H_2_O_2_), hydroxyl radicals (·OH-) and other forms. Oxidative stress is characterized by an imbalance in the action of intracellular antioxidants and pro-oxidants leading to massive production of ROS and consequent cell damage (Fatima et al., 2011). The disruption of redox homeostasis leads to a high production of ROS, which results in cellular damage (Fatima et al., 2011). The plant cell ROS system contains a variety of antioxidant enzymes such as catalase (CAT), superoxide dismutase (SOD) and peroxidase (POD), SOD catalyzes the reaction of ·O2- to produce H_2_O_2_, while CAT scavenges H_2_O_2_. POD catalyzes the H_2_O_2_-dependent oxidation of phenolic and other substrates, concomitantly reducing H_2_O_2_ to water. Polyphenol oxidase (PPO) can oxidize phenolics to produce quinones. The interaction of the three enzymes, SOD, POD and CAT can effectively regulate SE and development (Peng et al., 2020). As one of the decomposition products of cell membrane lipid peroxidation, malondialdehyde (MDA) levels represent degrees of cell membrane peroxidation and damage (Miller et al., 2008). However, the effect of ROS and MDA on the ability of blue spruce to mature somatic embryos has not been reported.

Phytohormone balance is critical for both short-term and long-term plant developmental processes and plant responses to environmental cues (Nambara et al., 2010). During SE, an important prerequisite for the conversion of somatic cells into embryonic cells is the induction of cell differentiation by *in vitro* culture and the application of appropriate plant growth regulators (PGRs). It is well known that the application of PGRs has an important effect on the process of SE (Gao et al., 2020a, b; Park et al., 2010). Addition of auxin and cytokinin in culture media will induce the proliferation of pro-embryogenic masses (PEMs), while addition of abscisic acid (ABA) induces the production of SEs (Filonova et al., 2000; Reeves et al., 2017; Lelu-Walter et al., 2008). Changes in levels of endogenous hormones are regulated by phytohormone signal transduction and phytohormone synthesis genes. The interactions and balance between different endogenous hormones affect the SE process, and their contents and ratios influence and regulate SE differentiation and development (Ruduś et al., 2009). Currently, there is no report available on the differences in the content of endogenous hormones during the maturation period and in different types of cell lines in blue spruce.

We have previously conducted a series of studies on SE in conifers. In *Picea pungens* (blue spruce), we optimized systems for embryogenic callus induction, SE maturation, and cryopreservation (Cao and Gao, 2022) and investigated the effects of plant growth regulators (e.g., 2,4-D) and sucrose on enhancing the efficiency and quality of embryogenic callus proliferation (Gao et al., 2023). Moreover, we demonstrated that exogenous glutathione promotes embryogenic cell proliferation and embryo development by regulating intracellular redox balance and endogenous hormone levels (Gao et al., 2024). For *Pinus koraiensis*, we optimized SE embryo maturation protocols (Peng et al., 2021) and identified morphological and physiological markers for the early screening of cell lines with high embryogenic potential, highlighting the critical roles of carbohydrate metabolism and redox homeostasis in embryo maturation (Peng et al., 2022). Our previous studies also revealed significant differences in embryo maturation capacity among different blue spruce cell lines (Gao et al., 2023). In this study, we systematically characterized four blue spruce cell lines differing in somatic embryo maturation capacity, with a focus on key physiological parameters associated with SE maturation. The resulting data provide a basis for optimizing the maturation medium and offer a framework for subsequent investigations into the molecular mechanisms governing SE formation in blue spruce.

## 2 Materials and methods

### 2.1 Plant material

Mature seeds of blue spruce were collected in 2008 from a tree (denoted as “2” owned by Sheffield’s Seed Company, New York, USA. The seeds were bought from the owner in 2011 and were stored at -40°C since then.

### 2.2 Establishment of embryogenic callus cultures

Seeds were rinsed under running water for 18 h, surface-sterilized with 75% medical- grade ethanol for 30 s and rinsed 3–5 times with sterile distilled water (1 min per rinse). Subsequently, seeds were sterilized with 4.2% sodium hypochlorite (NaClO; effective chlorine concentration: 4%; (Enshi Reagent, Hubei, China) for 15 min and rinsed again 3–5 times with sterile distilled water (1 min per rinse). Under sterile conditions, seed coats and endosperm were carefully removed, and the zygotic embryos were excised and cultured on EC induction medium. The medium consisted of full-strength of modified Litvay’s medium (mLV) (Litvay et al., 1985; Klimaszewska and Park, 2001), supplemented with 2.0 mg L^-1^6-BA (Sigma-Aldrich, Missouri, USA), 4.0 mg L^-1^ 2,4-D (Sigma-Aldrich, Missouri, USA) and 0.5 g L^-1^ L-glutamine (Sigma-Aldrich, Missouri, USA), with addition of, 0.8 g L^-1^ casein hydrolysate (Sigma-Aldrich, Missouri, USA) and 10 g L^-1^ sucrose (Sigma-Aldrich, Missouri, USA) (Cao and Gao, 2022) as well as 4 g L^-1^ gellan gum (Sigma-Aldrich, G1910-1KG, Missouri, USA). One hundred embryos were used in this study. Ten embryos were placed per Petri dish (90 mm diameter x 20 mm depth) (Shanghai Graft Horticultural Products Co., Ltd., Shanghai, China) for inducing EC cell lines. The embryo culture was maintained on the same medium in darkness during the whole EC induction period.

### 2.3 Proliferation of EC cell lines

Four stably proliferated EC cell lines (201, 202, 211, 226) were transferred to proliferation medium (PM), which consisted of full-strength mLV supplemented with 4.0 mg L^-1^ 2,4-D, 1.0 mg L^-1^ BA, 0.5 g L^-1^ glutamine, 0.8 g L^-1^ casein hydrolysate, 10 g L^-1^ sucrose and 4 g L^-1^ gellan gum. Cultures were maintained in darkness, and the medium was renewed every two weeks. For each EC cell line, 8 Petri dishes were cultured.

### 2.4 Somatic embryo culture and sample collection

EC samples (80 mg per cell line) were collected to determine their initial fresh weights. Subsequently, 4 mL of liquid PM medium without plant growth regulators were added. The cultures were shaken vigorously to disperse the cells and then filtered using a Büchner funnel. The EC cells were transferred onto 8-cm filter papers placed in Petri dishes containing somatic embryo maturation (SEM) medium and incubated in the dark for 8 weeks. The SEM medium consisted of full-strength mLV medium, supplemented with 13.22 mg L^-1^ ABA (Sigma-Aldrich, Missouri, USA), 0.5 g L^-1^ L-glutamine, 0.8 g L^-1^ casein hydrolysate, 30 g L^-1^ sucrose, 6.5 g L^-1^ gellan gum and 1 g L^-1^ activated charcoal.

For cytological, physiological and biochemical analyses, SE cultures were sampled on day 0 (proembryogenic masses stage), 15 (late-stage SE development), 30 (cotyledon-stage) and 45 (mature stage), with 12 g of tissue collected per sample. All samples were immediately frozen in liquid nitrogen and stored at -80°C until further use, following the protocol of Cao and Gao (2022).

### 2.5 Somatic embryo counting and morphological analysis

At day 60, the SEs were counted after staining, and embryo morphology was examined using a stereomicroscope (OLYMPUS SZX 7, Japan) equipped with a digital camera (Canon DS126271, Japan). For staining, fresh SEs were placed on a glass slide, followed by staining with 0.1% carmine solution for 10 minutes under a coverslip. To ensure uniform cell spreading, the coverslip was gently tapped with the flat end of a pencil. Observations and image acquisition were carried out using an optical microscope (OLYMPUS CX 31, Japan) equipped with a camera (Canon DS126271, Japan). The experiment was conducted in four independent biological replicates.

### 2.6 Germination test of somatic embryos

SE germination tests were initiated on day 45. SE samples were first dried in a 6-well cell culture plate (Corning-Costar 3516; Corning Incorporated, New York, USA). Two layers of dry, sterile filter paper were placed in three wells, on which SEs were positioned, while the remaining three wells contained sterile water to maintain humidity. Plates were sealed with Parafilm (Pechiney Plastic Packaging, Chicago, USA) and incubated in darkness at 4°C for 14 days. Following desiccation, SEs were transferred to germination medium consisting of full-strength mLV, supplemented with 2 g L^-1^ activated charcoal, 10 g L^-1^ sucrose and 4 g L^-1^ Gelrite. Cultures were maintained in darkness at 23°C for 7 days, followed by 14 days under light conditions of 8 h photoperiod with a light intensity of 100 μmol m^-2^ s^-1^ under cool white, fluorescent tubes. Germinated SEs were then transferred to culture flasks (Greiner Bio – One, Kremsmünster, Germany) for continued growth. After 3 months, plantlets were transplanted into a greenhouse and acclimatized in a substrate composed of soil, vermiculite and perlite at a ratio of 3:1:1.

### 2.7 Quantification of storage components

#### 2.7.1 Total protein

Soluble TP content was determined using the TP A045-2–2 kit; Nanjing Jianjian Bioengineering Institute (Nanjing, China) according to the manufacturer’s instructions. In brief, embryo tissues of 0.2 g along with 0.8 mL of the extraction solution were homogenized in mortar on ice. The homogenates were transferred into centrifuge tubes and centrifuged at 1800 g for 10 min. The supernatant was diluted 5 times with distilled water. The diluted supernatant of 0.05 mL along with 3 mL of Coomassie brilliant blue solution were mixed vigorously and kept at room temperature for 10 min. The mixture was then measured on a UV 2800S Dual-Beam Ultraviolet-Visible spectrophotometer (Shanghai Yuanxi Instrument Co., Ltd., Shanghai, China) at 595 nm. Each treatment was conducted with 3 biological replicates. Protein content was calculated using the following formula.


$$\text{Protein content}(\text{mg}/\text{g})=\frac{\text{Asample}-\text{Ablank}}{\text{Astandard}-\text{Ablank}}\times \text{C}\times \text{F}\times \text{V}÷\text{W}$$

Where Asample = absorbance of sample; Ablank = absorbance of blank; Astandard = absorbance of standard protein (BAS); C = BAS concentration (0.524 g/L); F = extract dilution factor prior to assay; V = total extract volume; W = sample fresh weight (g).

#### 2.7.2 Soluble sugar

SS content was determined using the SS A145-1–1 kit; Nanjing Jianjian Bioengineering Institute (Nanjing, China) according to the manufacturer’s instructions. Embryo tissues of 0.2 g along with 0.8 mL of the extraction solution were homogenized in mortar on ice, followed by boiling in water bath for 15 min. After cooling down, the homogenates were centrifuged at 1800 g at room temperature for 10 min. The supernatant was then diluted 20 times with distilled water. The diluted supernatant of 200 μLwas mixed with 100 μL of substrate solution and 1000 μL of concentrated sulfuric acid sequentially in a tube. The tubes were then boiled in water bath for 10 min. After cooling down with running water, 200 μL of the supernatant was added into a 96-well cell plate for measuring sugar content. The OD values were obtained at 620 nm using an enzyme counter (BIOTEK, Vermont, USA). The experiment was conducted with 3 biological replicates. Soluble sugar content was calculated using the following formula:


$$\text{Soluble sugar content}(\text{mg}/\text{g})=\frac{\text{Asample}-\text{Ablank}}{\text{Astandard}-\text{Ablank}}\times \text{C}\times \text{F}\times \text{V}÷\text{W}$$

where Asample = absorbance of sample; Ablank = absorbance of blank; Astandard = absorbance of standard sugar (glucose); Cstandard = glucose concentration (0.1 mg/mL); F = Extract dilution factor prior to assay; V_Extract_ = total extract volume (mL); W = sample fresh weight (g).

#### 2.7.3 Starch

ST content was determined using the Starch BC0705 kit; Beijing Solepol Technology Co. Ltd (Beijing, China). Embryo tissues of 0.2 g along with 1.8 mL of reagent 1 were homogenized in mortar on ice. The homogenates were transferred to 2 ml centrifuge tubes (Eppendorf, Hamburg, Germany) and extracted in water bath at 80°C for 30 min, followed by centrifugation at 2800 g at room temperature for 5 min. The supernatant was discarded. Double-distilled water of 0.5 mL was added in the tube and boiled in water bath to paste for 15 min. After cooling down, 0.35 mL of reagent II was added and extracted at room temperature for 15 min with shaking 3–5 times. Thereafter, 0.85 mL of double-distilled water was added into the sample solution, mixed vigorously, and centrifuged at 2800 g for 10 min at room temperature, and the supernatant was diluted 7 times with distilled water. For the assay, 50 μL of diluted sample solution along with 250 μL of reagent solution were mixed in a tube and warmed in water bath at 95°C for 10 min. After cooling down to room temperature, 200 μL was added into a 96-well cell plate for measurement at 620 nm using an enzyme counter (BIOTEK, Vermont, USA). Each treatment was conducted with 3 biological replicates. Starch content was calculated using the following formula:


$$\text{Starch content}(\text{mg}/\text{g)}=\frac{(\text{Asample}-\text{Ablank})-0.0616}{3.9324}÷\text{F}\times \text{V}÷\text{W}÷\text{d}$$

where Asample = absorbance of sample; Ablank = absorbance of blank; 3.9324 and 0.0616 were from the formula y = 3.9324 x + 0.0616, obtained from the glucose standard curve, in which y = absorbance of glucose; F = extract dilution factor prior to assay; V = total extract volume; W = sample fresh weight (g); d = constant (1.11) used for conversion from glucose to starch.

### 2.8 Measurement of reactive oxygen species-related enzyme activities

#### 2.8.1 Superoxide dismutase activity

SOD activity was determined using the SOD BC0175 kit; Beijing Solebo Technology Co. (Beijing, China). Embryo tissues of 0.2 g along with 1.8 mL of reagent 1 were homogenized in mortar on ice. The homogenates were centrifuged at 3600 g at 4°C for 10 min. Prior to measurement, regents 1, 3 and 5 were placed in 25°C water bath for at least 10 min. The supernatant of 18 µL was mixed with 45 μL reagent 1, 2 μL reagent 2, 35 μL reagent 3, 90 μL water and 10 μL reagent 5 according to the manufacturer’s instructions. The mixtures were placed in 35°C water bath for 30 min. The samples (200 μL for each) were then loaded in a 96 cells plate and OD values were obtained at 560 nm using an enzyme counter (BIOTEK, Vermont, USA). The calculation of SOD was according to the manufacturer’s instructions. Each treatment was conducted with 3 biological replicates.

#### 2.8.2 Catalase activity

CAT activity was determined using the CAT BC0205 kit; Beijing Solebo Technology Co. (Beijing, China). Embryo tissues of 0.1 g along with 1.9 mL of extraction buffer were homogenized in mortar on ice. The homogenates were centrifuged in 2 mL tube at 3600 g at 4°C for 10 min and supernatants were collected. The reagent was placed in 25°C water bath for at least 10 min. For measurement, 10 μL supernatant and 190 μL reagent was added in a 96-cells plate according to the manufacturer’s instructions and mixed well. The OD value was obtained at 240 nm using an enzyme counter (BIOTEK, Vermont, USA), and the CAT activity was then calculated according to the manufacturer’s instructions. Each treatment was conducted with 3 biological replicates.

#### 2.8.3 Peroxidase activity

POD activity was determined using the POD BC0095 kit; Beijing Solebo Technology Co. (Beijing, China). Embryo tissues of 0.1 g along with the extraction solution of 1.8 mL were homogenized in mortar on ice, followed by centrifugation at 3600 g at 4°C for 10 min. Prior to measurement, reagents 1: 2: 3 were mixed with water in 4:1:1:2 ratio and the mixture were placed in 25°C water bath for at least 10 min. The supernatant of 5 μL was mixed with 240 μL of the mixed reagents according to the manufacturer’s instructions. The mixtures were then loaded on a 96 cells plate. The OD values were collected at 470 nm using an enzyme counter (BIOTEK, Vermont, USA). One unit of enzyme viability was defined as a change of 0.005 in A470 per g of tissue per minute in each mL of the reaction system according to the manufacturer’s instructions. Each treatment was conducted with 3 biological replicates.

#### 2.8.4 Polyphenol oxidase activity

PPO activity was determined using the PPO BC0195 kit; Beijing Solebo Technology Co. (Beijing, China). Embryo tissues of 0.1 g along with 0.6 mL of extraction solution were homogenized in mortar on ice, followed by centrifugation at 3600 g at 4°C for 10 min. The supernatant was collected and placed on ice for further use. For measurement, 50 μL supernatant was mixed with 200 μL reagent 1, 50 μL reagent 2. The mixtures were placed on 25°C water bath for 10 min, followed by boiling for 10 min according to the manufacturer’s instructions. The mixtures were mixed vigorously and centrifuged at 2250 g at 4°C for 10 min. Supernatants of 200 μL were then loaded on a 96 cells plate for measuring enzyme activities at the OD value of 410 nm using an enzyme counter (BIOTEK, Vermont, USA). Each treatment was conducted with 3 biological replicates.

### 2.9 Malondialdehyde

MDA content was determined using the MDA A003–1 kit; Nanjing Jiancheng Bioengineering Institute (Nanjing, China). Embryo tissues of 0.2 g along with 0.6 mL of PBS solution (0.1 mol L^-1^) were homogenized in mortar on ice. After centrifugation at 1800 g for 10 min, the supernatant of 80 μL and 80 μL of Reagent I were mixed thoroughly in a glass tube, followed by addition of 1200 μL of Reagent II and 400 μL of Reagent III according to the manufacturer’s instructions. The tubes were incubated in a boiling water bath for 40 minutes, followed by cooling down under running water and centrifuged at 1800 g for 10 minutes. Supernatants of 200 μL were loaded in a 96-well cell plate and OD values at 532 nm were obtained using an enzyme counter (BIOTEK, Vermont, USA) according to the manufacturer’s instructions. Each treatment was conducted with 3 biological replicates.

### 2.10 Quantification of endogenous hormone levels

Endogenous IAA, ABA, GA and ZR contents were determined by Enzyme-linked Immunosorbent Assays (ELISA) using the Mlbio ELISA kits; Shanghai Enzyme-Link Bio-technology Co., Ltd. (Shanghai, China). Embryogenic tissues of 0.5 g along with 4.5 mL of 0.01 mol L^-1^ phosphate buffer (pH=7.2-7.4) were homogenized in mortar on ice. After centrifugation at 1800 g for 20 min at room temperature, the supernatant was taken and stored on ice or fridge. The extracts and reagents were added according to the manufacturer’s instructions to determine the contents of endogenous IAA, ABA, GA and ZR. Each treatment was conducted with 3 biological replicates.

### 2.11 Statistical analysis and graphics

As stated in material section, all experiments or biochemical analyses were repeated and the number of biological replicates varied among the different analyses, which are specified in each section as stated above. SPSS 23 software was used for ANOVA and multiple comparison tests using the LSD method. SigmaPlot 12.0 software was used for graphing.

## 3 Results

### 3.1 EC induction and comparison of SE capacity among different EC cell lines

Out of 100 seed explants, 53 seeds resulted in EC formation, giving the induction rate of 53%. From the EC cell lines, the best four were used in further studies.

**Figure 1:**
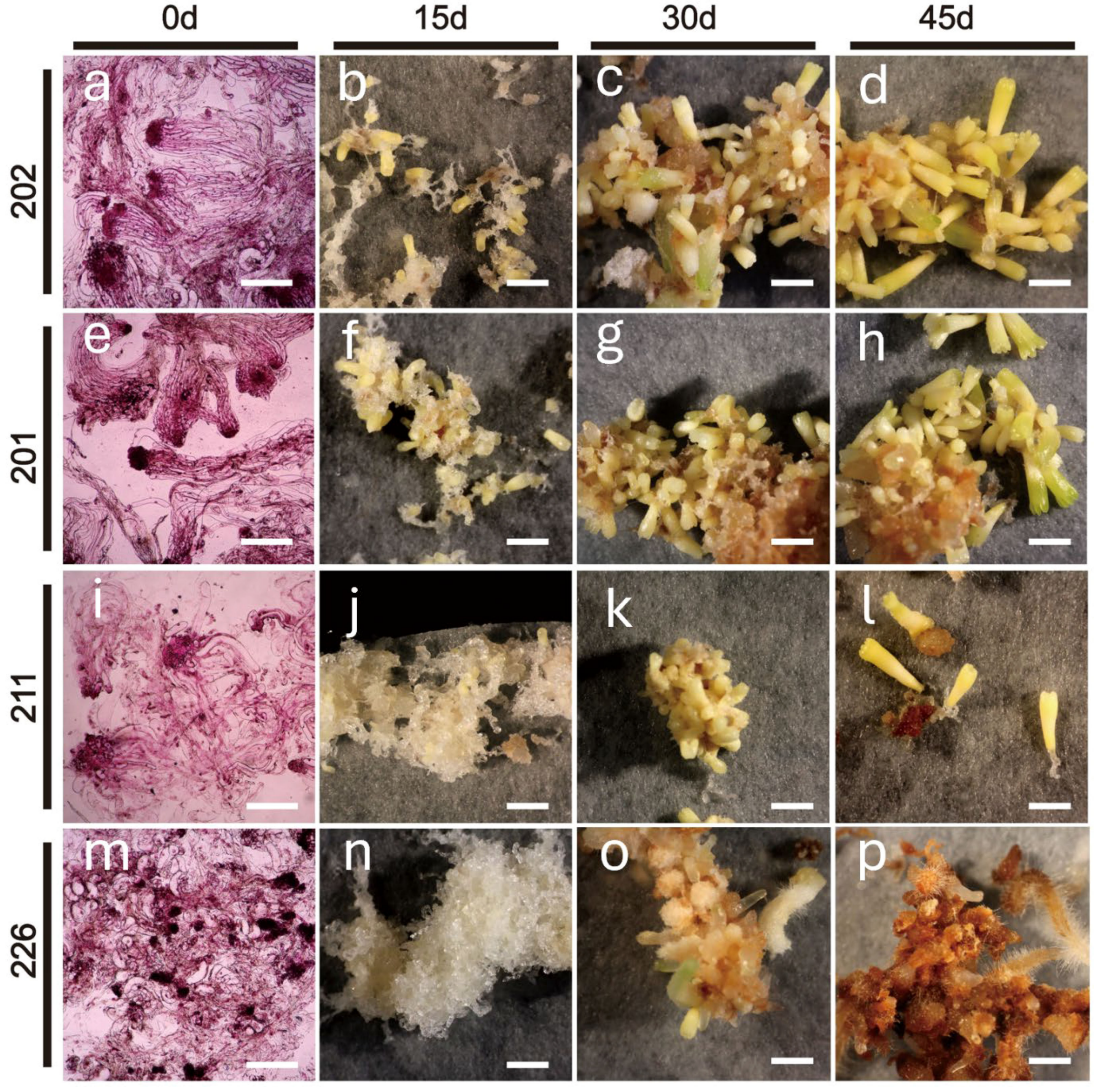
Different EC cell lines from the same tree of blue spruce have shown different somatic embryogenesis and SE maturation capacities during different SE developmental stages under the same *in vitro* culture conditions observed under microscope. **(A, E, I, M)** = EC cultures or embryos stained with 0.1% carmine solution at day 0 for cell lines 202, 201, 211, 266, respectively. **(B–D, F–H, J–L, N–P)** represent EC cultures or embryos on day 15, 30 and 45 for cell lines 202, 201, 211 and 266, respectively. Scale bar: **(A, E, I, M)**, 100 nm; **(B–D, F–H, J–L, N–P)**, 0.5 cm.

All four cell lines maintained stable proliferation on the PM medium; however, their SE maturation capabilities differed significantly. After 15 days of maturation culture, cell line 202 yielded the highest number of late-stage SEs, reaching 1,625 SEs g^-1^ FW (**Figure 1B**), followed by Cell line 201 with 1,350 SEs g^-1^ FW (**Figure 1F**). In contrast, cell line 211 showed slower SE development, yielding only 325 SEsg^-1^ FW (**Figure 1J**), while cell line 226 exhibited only callus proliferation and failed to produce any mature SE (**Figure 1N**).

Cytological analysis revealed distinct differences in internal callus cell structures of the four cell lines from day 0 on PM medium (**Figures 1A, E, I, M**). Cell line 202, which exhibited the highest embryogenic potential, contained an abundant number of early SEs characterized by large embryonic heads and well-defined structures (**Figure 1A**). Cell line 201 (**Figure 1E**) showed a similar but less pronounced pattern, producing fewer SEs and smaller embryonic heads than 202 (**Figure 1A**). Cell line 211 formed only a limited number of poorly structured SEs (**Figure 1I**), whereas cell line 226 (**Figure 1M**) failed to produce early SEs displaying numerous scattered cell clusters instead. After 15 days of culture on the PM medium, morphological differences became more pronounced: cell lines 202 and 201 had already produced many late-stage SEs (**Figures 1B, F**). By day 30, the SE-competent lines (202, 201, and 211) formed cotyledonary SEs (**Figures 1C, G, K**). At day 45, mature SEs formed were as follows: 1,625 ± 82 SEs g^-1^ FW for 202 (**Figure 1D**); 1,350 ± 178 Ses g ^-1^ FW for 201 (**Figure 1H**); and 325 ± 22 SEs g^-1^ FW for 211 (**Figure 1I**). Cell line 226 (**Figure 1P**) continued to show only proliferation with no SE development.

### 3.2 Germination and planting

**Figure 2:**
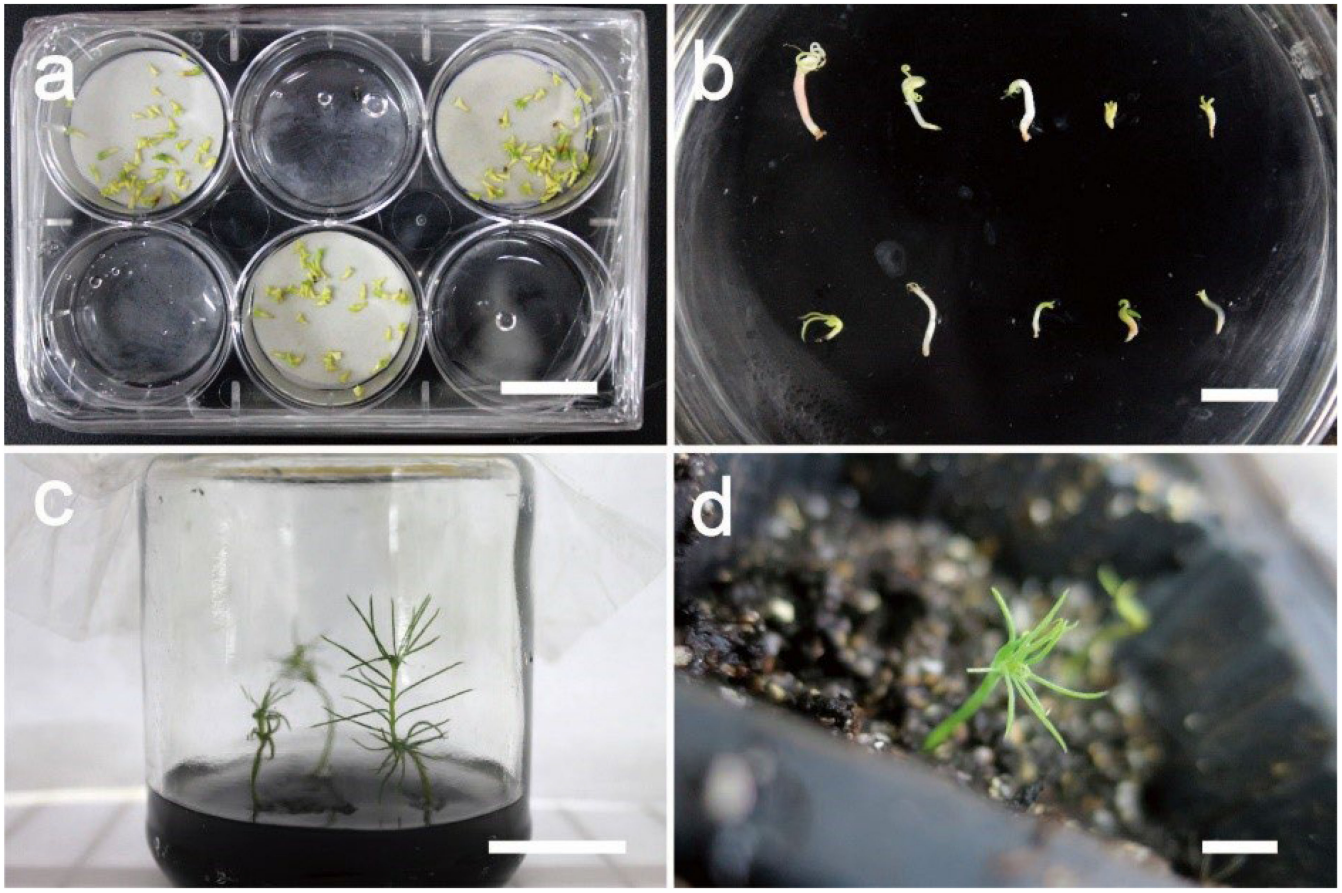
*In vitro* desiccation, germination and growth as well as ex vitro establishment of somatic embryos of cell line 202 of blue spruce. **(A)** desiccation treatment of mature SEs; **(B)** SE germination; **(C)** Germinated plantlets from 3-month-old SEs; **(D)** Plantlets established well in soil in greenhouse. Scale bar: **(A)** 2 cm; **(B)** 1 cm; **(C)** 5 cm; **(D)** 4 cm.

To obtain healthy plantlets from SEs, desiccation is needed before the SEs can germinate (**Figure 2A**). The germination test showed SEs germinated normally on the germination medium (**Figure 2B**) and the germinated SEs grew vigorously on a large culture container (**Figure 2C**) after 3 months. The plantlets were well established ex vitro on the substrate (**Figure 2D**).

### 3.3 Changes in the levels of storage compounds

#### 3.3.1 Total protein

**Figure 3:**
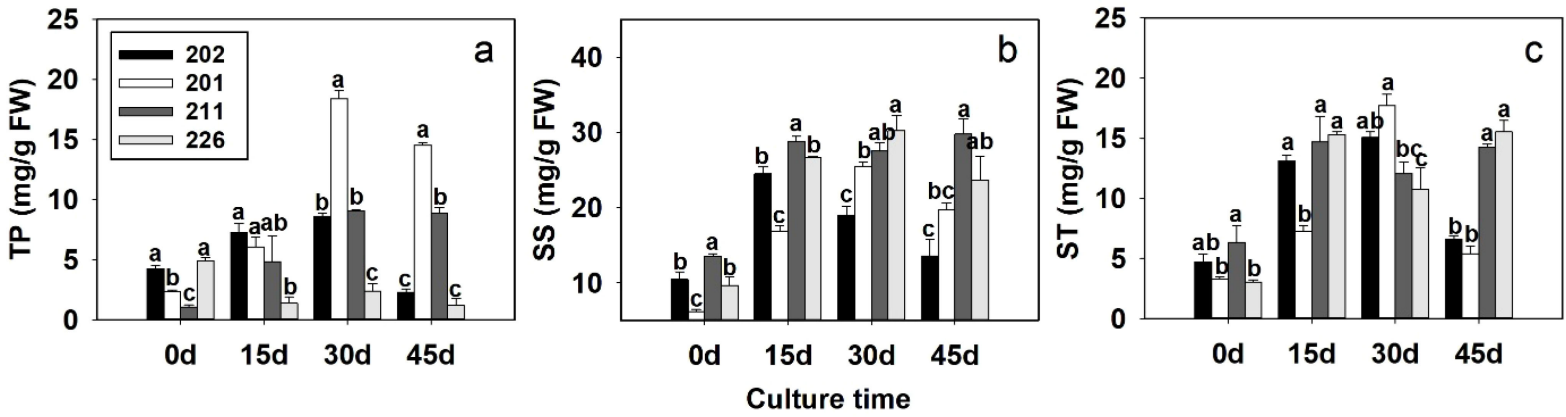
Changes in content of total protein (TP) **(A)**, soluble sugar (SS) **(B)** and starch (ST) **(C)** in during different developmental stages of somatic embryos of different cell lines of blue spruce with different somatic embryogenetic capacities. Different letters above the bars indicate significant differences at *p* < 0.05 among the cell lines at the same culture stage. Error bars represent ± SD.

At day 0, the total soluble TP content varied significantly among the four cell lines. Cell lines 202 and 226 exhibited similar TP levels, both of which were significantly higher than those observed in cell lines 201 and 211 (**Figure 3A**). By days 15 and 30, the TP levels in cell lines 202, 201, and 211 increased and became comparable in most cases, all significantly higher than the TP level in cell line 226. However, by day 45, TP levels in cell lines 202 and 226 declined and were significantly lower than those in cell lines 201 and 211.

In terms of overall trends, cell lines 202, 201, and 211 showed a general increase in TP levels throughout the culture period, whereas cell line 226 displayed a marked and continuous decline (**Figure 3A**).

These findings suggest a positive correlation between SE capacity and TP content, particularly during the early stages of culture (up to day 30). The sustained reduction in TP levels observed in cell line 226 may be linked to its inability to undergo somatic embryogenesis.

#### 3.3.2 Soluble sugar content

At day 0, SS levels varied significantly among the cell lines, except between 202 and 226, which showed no significant difference. Overall, SS content increased sharply across all cell lines at days 15, 30, and 45. Notably, in highly embryogenic cell lines 202 and 201, SS levels began to decline as SEs s matured, especially by day 45 (**Figure 3B**). This suggests that SS accumulation and subsequent reduction are closely linked to the development and maturation of SEs.

#### 3.3.3 Starch content

ST content generally increased in all cell lines with extended culture duration. However, no significant differences were observed between high (202, 201) and low (211, 226) embryogenic cell lines during most of the culture period. By day 45, ST levels in the highly embryogenic lines were significantly lower than in the less embryogenic ones, despite remaining slightly above the initial values (**Figure 3C**).

### 3.4 ROS-related enzyme activities

#### 3.4.1 Catalase activity

**Figure 4:**
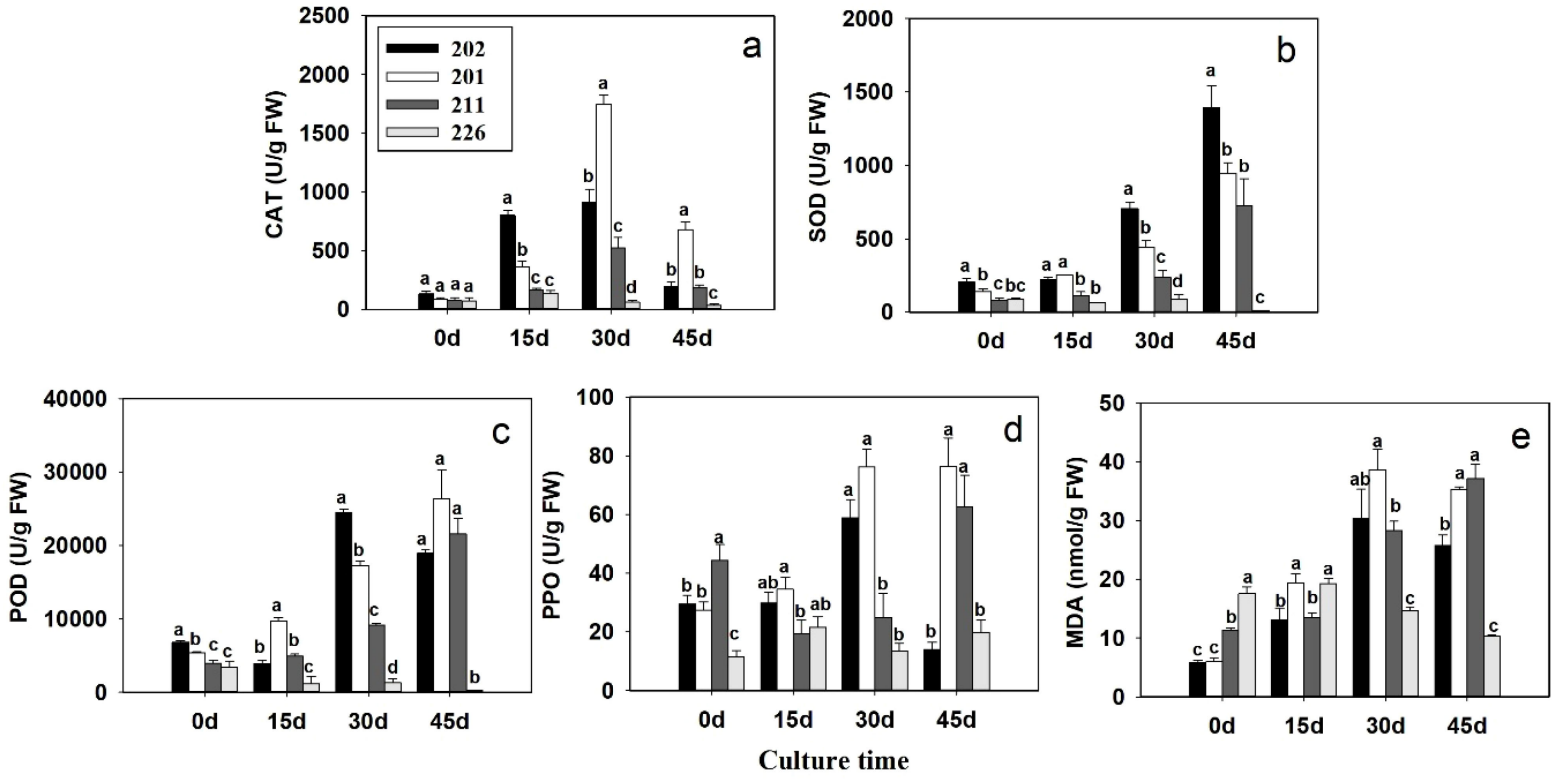
Changes in content of catalase (CAT) **(A)**, superoxide (SOD) **(B)**, peroxidase (POD) **(C)**, polyphenol oxidase (PPO) **(D)**, and malondialdehude (MDA) **(E)** during different developmental stages (day 0, 15, 30 and 45) of different cell lines of blue spruce with different somatic embryogenetic abilities. Different letters above bars represent significant differences at *p* < 0.05. Error bars represent ± SD.

At day 0, no significant differences in CAT activity were observed among the four cell lines (**Figure 4A**). By day 15, CAT activity had increased significantly, particularly in the two cell lines with higher SE capacity (202 and 201) and continued to rise by day 30. Cell line 211, with lower SE capacity, also showed elevated CAT activity compared to non-embryogenic line 226. At day 45, CAT activity declined across all cell lines but remained above baseline levels in cell lines 202, 201, and 211, suggesting sustained antioxidant response during SE maturation.

#### 3.4.2 Superoxide dismutase activity

SOD activity increased slightly by day 15, followed by a sharp rise at day 30, peaking at day 45 in the SE-competent cell lines (202, 201, and 211). In contrast, cell line 226 maintained consistently low SOD activity throughout the culture period (**Figure 3B**). Notably, at day 45, the SOD activity in cell line 202 reached 1,389.37 U/g FW, significantly higher than those in the other lines, particularly compared to the minimal activity in cell line 226 (12.68 U/g FW).

#### 3.4.3 Peroxidase activity

At day 0, POD activity was significantly higher in cell lines 202 and 201 compared to cell lines 211 and 226 (**Figure 4C**). As the culture progressed, POD activity varied among the lines. Cell lines 201, 202 and 211 consistently showed the highest activity, while cell line 226 remained the lowest. A sharp increase in POD activity was observed at days 30 and 45 in cell lines 202, 201, and 211. For instance, by day 30, POD activity reached 24,407.88 U/g FW in cell line 202, 17,240.16 U/g FW in cell line 201, and 9,125.76 U/g FW in cell line 211, whereas cell line 226 reached only 1,305.36 U/g FW.

#### 3.4.4 Polyphenol oxidase activity

At day 0, PPO activity was significantly higher in cell lines 202, 201, and 211 compared to cell line 226 (**Figure 4D**). The highest early PPO activity was recorded in cell line 211. A dramatic increase occurred at day 30 in cell lines 202 and 201, reaching 24,407.88 U/g FW and 17,240.16 U/g FW, respectively, while cell lines 211 and 226 showed much lower levels. At day 45, PPO activity dropped sharply in cell line 202 (13.92 U/g FW), while remaining relatively high in cell lines 201 and 211. Cell line 226 showed consistently low and stable PPO activity throughout.

### 3.5 Malondialdehyde content

At day 0, MDA content was lowest in cell lines 202 and 201 and highest in cell line 226 (**Figure 4E**). By day 15, MDA levels increased in cell lines 202 and 201, while remaining unchanged in cell lines 211 and 226. At days 30 and 45, MDA content rose significantly in cell lines 202, 201, and 211, but decreased in cell line 226.

### 3.6 Changes in hormone content

#### 3.6.1 IAA

**Figure 5:**
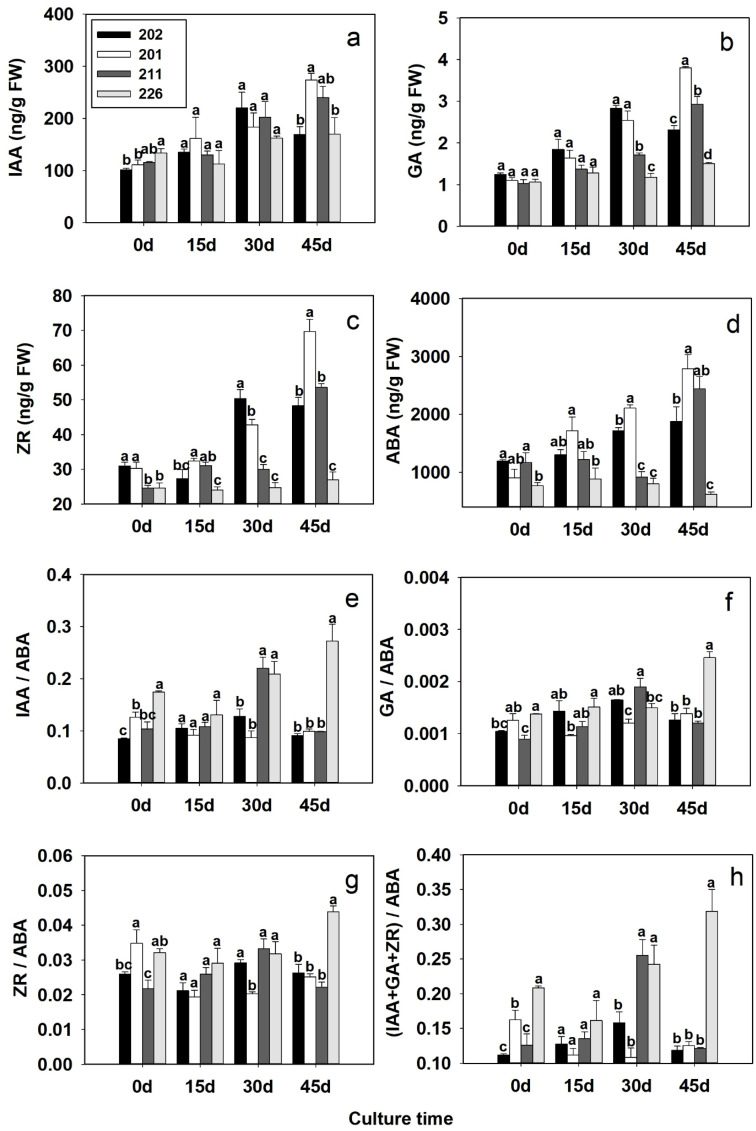
Changes in endogenous hormone (IAA **(A)**, GA **(B)**, ZR **(C)** and ABA **(E)**) contents and hormone ratios (IAA/ABA **(E)**; GA/ABA **(F)**; ZR/ABA **(G)**; (IAA+GA+ZR)/ABA **(H)**) in somatic embryos at different developmental stages of four blue spruce cell lines with different somatic embryogenetic capacities. Different letters above the bars indicate significant differences at *p* < 0.05 among the cell lines at the same culture stage. Error bars represent ± SD.

At day 0, the IAA content was significantly higher in non-embryogenic cell line 226 compared to cell lines 202 and 201. However, no significant differences were observed among all lines at day 15 and 30, while cell line 201 showed a notably higher IAA level than cell lines 202 and 226 at day 45 (**Figure 5A**).

#### 3.6.2 GA

There were no significant differences in GA content among the cell lines up to day 15 (**Figure 5B**). At day 30, GA levels were significantly higher in cell lines 202 and 201 compared to cell lines 211 and 226. By day 45, significantly higher GA levels were observed in cell lines 201, 202 and 211 compared to cell line 226, suggesting a stronger role of GA in late-stage SE maturation.

#### 3.6.3 ZR

ZR content was consistently higher in cell lines 202 and 201 than in cell lines 211 and 226 during the whole experimental period, except for some irregular changes at day 15 (**Figure 5C**). A sharp increase occurred at days 30 and 45 for cell lines 202 and 201. By day 45, ZR content peaked in cell line 211, while cell line 226 maintained the lowest levels throughout the culture period.

#### 3.6.4 ABA

At day 0, ABA content was significantly higher in cell lines 202 and 211 than in cell line 226 (**Figure 5D**). At day 15, only cell line 201 showed significantly higher ABA content than cell line 226. By day 30, ABA content remained significantly higher in the SE-competent cell lines 202 and 201 compared to cell lines 211 and 226, while at day 45 ABA content was significantly higher in cell lines 202, 201 and 211 than that in cell line 226.

#### 3.6.5 Changes in IAA/ABA, GA/ABA, ZR/ABA and IAA+GA+ZR/ABA ratios

At day 45, all four hormone ratios were significantly higher in cell line 226 compared to the other cell lines (**Figures 5E–H**). This trend was consistent across IAA/ABA, GA/ABA, ZR/ABA, and (IAA+GA+ZR)/ABA ratios, especially at later stages of culture. Notably, cell line 201 also showed a higher IAA/ABA ratio than cell line 202.

## 4 Discussions

Somatic embryogenesis (SE) in conifers, including blue spruce (*Picea pungens*), is a complex developmental process coordinated by morphological progression, metabolic reprogramming, redox regulation, and hormonal control (Aitken-Christie et al., 1995; Fraga et al., 2023; Gao et al., 2023; Von Arnold et al., 1996). Although SE shares feature with zygotic embryogenesis, its *in vitro* formation is strongly genotype dependent and stress sensitive (Méndez-Hernández et al., 2019). Comparative analysis of four blue spruce cell lines with distinct embryogenic capacities in this study revealed that SE competence arises from the integration of morphogenetic potential, oxidative homeostasis, reserve accumulation, and hormone-mediated regulation.

### 4.1 Morphological and physiological determinants of SE

Progression through the proembryogenic mass (PEM) stages (Filonova et al., 2000) is a key determinant of somatic embryogenesis (SE) capacity. Highly embryogenic lines (202, 201) advanced to PEM III and formed early bipolar embryos, whereas line 226 remained arrested at PEM II, exhibiting signs of oxidative stress and cellular damage. This oxidative stress likely resulted from elevated ROS levels originating from culture conditions: 2,4-D activates NADPH oxidase to generate superoxide anions, BA enhances mitochondrial respiration and ROS production, and sucrose-induced osmotic stress further amplifies oxidative imbalance. Therefore, morphogenetic differentiation, rather than callus proliferation, determines SE success, consistent with previous findings in larch and spruce (Bozhkov et al., 2010; Savane et al., 2023).

Embryogenic lines (202 and 201) also exhibited early and coordinated accumulation of soluble sugars and starch, providing both metabolic energy and structural carbon for embryo development. Concurrent increases in total protein (TP) reflected the synthesis of storage proteins and antioxidant enzymes such as SOD and CAT (Businge et al., 2012; Hazubska-Przybył et al., 2022). In contrast, the non-embryogenic line 226 showed limited reserve buildup and weak stress adaptation, underscoring the link between metabolic readiness and embryogenic potential.

### 4.2 Reactive oxygen species metabolism and redox control

ROS act as essential developmental signals during SE, but their levels must remain tightly controlled. In this study, SOD activity in embryogenic lines peaked around day 45, followed by increases in CAT and POD activity, forming a sequential “conversion–clearance” mechanism that maintained optimal ROS balance. This regulated redox environment promoted differentiation while preventing oxidative injury. In contrast, non-embryogenic lines exhibited insufficient antioxidant activity and excessive lipid peroxidation, indicating ROS imbalance. These findings are consistent with reports in *Pinus* and *Lycium barbarum* (Cui et al., 1999; Rahal et al., 2014). Moderate MDA accumulation in embryogenic lines supports the “ROS metabolic switch” hypothesis, wherein controlled ROS flux facilitates cellular reprogramming, while excessive or insufficient ROS suppresses SE (Cui et al., 1999; Fatima et al., 2011; Wu et al., 2020).

### 4.3 Hormonal regulation and crosstalk

Endogenous hormones closely interact with ROS metabolism to regulate SE progression (Méndez-Hernández et al., 2019; Chen et al., 2021). Auxin (IAA) increased during induction across all lines, but similar IAA levels in embryogenic and non-embryogenic lines suggest that responsiveness—rather than concentration—determines SE competence (Ayil-Gutiérrez et al., 2013; Kępczyńska and Kępczyńsk, 2023). Embryogenic lines also exhibited elevated gibberellin (GA) levels during maturation, supporting embryo elongation (Kim et al., 2019), and a moderate rise in zeatin riboside (ZR), facilitating cell proliferation and differentiation.

Abscisic acid (ABA) peaked at day 45 in embryogenic lines, coinciding with starch and protein accumulation. This suggests that ABA promotes reserve mobilization and desiccation tolerance (Yan & Chen, 2017). Elevated POD activity and moderate MDA levels during the ABA peak indicate functional ABA–ROS crosstalk, aligning antioxidant activity with developmental transitions. The low ABA and high GA/ABA ratios in line 226 disrupted this coordination, impairing maturation.

### 4.4 Integrated regulatory model

SE competence in blue spruce depends on the synchronization of morphological differentiation, energy reserve metabolism, ROS homeostasis, and hormonal signaling. Among these, ABA–ROS interplay appears central to integrating stress adaptation with developmental programming. Highly embryogenic lines maintain this network effectively, whereas non-embryogenic lines exhibit disrupted integration, poor metabolic balance, and limited morphogenetic progression.

Biochemical markers such as high TP content, elevated SOD and POD activity, and moderate MDA accumulation consistently indicated embryogenic potential, suggesting their utility for early screening of responsive cell lines. Manipulating hormonal ratios—particularly optimizing ABA relative to GA and IAA—and enhancing antioxidant capacity may further improve SE outcomes. Future work should explore the transcriptional and metabolic regulators mediating ABA–ROS interactions and reserve mobilization to inform rational optimization of culture systems for conifer SE.

## 5 Conclusions and future perspectives

Comparative analysis of four cell lines revealed clear differences in SE potential. Cell line 202 had the highest capacity, followed by cell line 201, whereas cell line 211 showed limited development, and cell line 226 failed to initiate embryogenesis. These findings highlight the importance of selecting high-performing cell lines such as 202 for efficient plant regeneration. Changes in total soluble protein content were associated with embryogenic potential. Embryogenic lines (202, 201, 211) showed an early increase in protein levels during culture, while the non-embryogenic 226 exhibited a decline, suggesting that protein accumulation may serve as a marker of embryogenic competence. Soluble sugar content increased throughout culture and was positively linked to somatic embryo development, while starch accumulation appeared less critical but may indicate utilization during later maturation stages. Higher CAT and SOD activities, particularly from day 15 to 45, were associated with successful embryo development, implying that effective ROS scavenging supports SE. Strong embryogenic cell lines exhibited higher POD and PPO activities and lower initial MDA levels, indicating that regulation of oxidative stress contributes to SE development. High SE potential in embryogenic lines correlated with elevated absolute levels of endogenous GA, ZR, and ABA, while the non-embryogenic cell line 226 displayed a high ratio of growth-promoting hormones to ABA but lower overall hormone concentrations, suggesting that both hormone balance and adequate hormone levels are essential for SE success.

Future research should focus on three key areas: 1) Molecular mechanisms: combining molecular markers with gene editing technologies to analyze how ROS (e.g. H_2_O_2_) regulate antioxidant enzyme genes (SOD, POD) and influence embryo maturation, thereby clarifying the functions of key regulatory genes; 2) Hormonal regulation: Applying multi-omics integration technology to construct synergistic regulatory networks of endogenous hormones (GA, IAA, ABA, etc.) across different SE maturation stages to elucidate the dynamic relationship between hormone balance and SE quality; 3) Field adaptability: Conducting long-term adaptability assessments of SE-derived plants under abiotic stresses (e.g. drought, low temperature, and salinity) to validate the correlation between physiological markers and actual stress physiological markers and actual stress tolerance. These studies would provide theoretical supports for the large-scale application of SE-derived coniferous plantlets, promoting their efficient adaptation in landscape and forestry production while contributing new insights into the foundational frameworks of plant SE development.

## Data Availability

The original contributions presented in the study are included in the article/supplementary material. Further inquiries can be directed to the corresponding authors.
